# Understanding the post-diagnostic support priorities of autistic adults in the United Kingdom: A co-produced modified Delphi study

**DOI:** 10.1177/13623613231196805

**Published:** 2023-09-30

**Authors:** Susanna Crowson, Daniel Poole, Kelly Scargill, Megan Freeth

**Affiliations:** 1 The University of Sheffield, UK; 2 Disability Sheffield, UK

**Keywords:** adults, co-design, health services, participatory, post-diagnostic

## Abstract

Post-diagnostic support for autistic adults in the United Kingdom is geographically inequitable and, in general, considered inadequate. This results in autistic adults facing unnecessary challenges and wide-ranging poor outcomes. A modified Delphi study sought to establish autistic adults’ priorities for optimal provision of post-diagnostic support. The study was co-designed with 10 autistic adults across a series of three online workshops. Forty-three autistic adults, who had received their diagnosis in adulthood, completed a series of three online questionnaires through which 153 support priorities were identified. In a fourth questionnaire round, completed by 139 autistic adults, 24 priorities reached or exceeded a consensus threshold of 80% agreement that the item specified an important element of post-diagnostic support. Participants ranked *access to support where they live*, *training of professionals*, *support to process the impact of a late diagnosis*, *use of their preferred mode of contact* and *an individualised support plan* as their key priorities for post-diagnostic support. The whole priority list provides a clear and practical articulation of what autistic adults want from post-diagnostic support. This should be used by policymakers and service providers in the United Kingdom to ensure that post-diagnostic support for autistic adults is fit for purpose.

**Lay abstract:**

Autistic adults in the United Kingdom report that support for themselves and their peers is not suitable for their needs. There has been an increase in adults receiving an autism diagnosis, which many have reported as having a positive impact on their lives. However, the lack of support and understanding after diagnosis, combined with long wait times for an assessment to obtain a diagnosis and to access follow-on support, is having a negative impact on people’s lives. This study took place to find out what support autistic people need and want after receiving their diagnosis. It was co-designed with a group of 10 autistic adults which means that the researchers and group members collaboratively designed the research. For the study, 43 autistic adults, diagnosed aged 18 or older, completed three questionnaires. A fourth questionnaire followed that was completed by 139 autistic people who received their diagnosis in adulthood. These questionnaires aimed to help people identify their own priorities when it came to the support they would have liked to receive after being given their autism diagnosis. Participants ranked *access to support where they live*, *training of professionals*, *support to process the impact of a late diagnosis*, *use of their preferred mode of contact* and *a personalised support plan* as their top priorities. This demonstrates that local support is highly valued by autistic adults, as are well-trained professionals who offer a range of contact options, support to process a late-in-life autism diagnosis and help to develop and implement support plans.

Autism is increasingly being recognised in adulthood. Recorded autism diagnoses rose from one to 20 per 100,000 adults in the United Kingdom over a recent 20-year period (1998 to 2018; Russell et al., 2022). Increase in adult diagnoses has been attributed to a broadened diagnostic criteria (Rutter, 2005) and improved understanding of autism presentations (Russell et al., 2022). Relatedly, local provision of adult autism diagnostic services has improved, with less than 50% of local authorities having an adult autism diagnostic service in 2009, compared with 93% in 2019 (National Autistic Society (NAS), 2019). While adult autism diagnosis is increasingly available and prevalent, autism in adult populations has been the focus of comparatively less research than autism in paediatric populations and adult services lag far behind those for children (Howlin, 2021). There is a clear need to more effectively understand the needs and perspectives of autistic adults for services to be appropriately designed and delivered, and thus better outcomes achieved.

In the United Kingdom, routes to diagnosis via the National Health Service (NHS) network of adult diagnostic services are heterogeneous and vary from one local authority to another (Crane et al., 2018). Despite the substantial regional variation, pathways generally involve referral to specialist autism or neurodevelopmental services where a diagnostic assessment for autism is undertaken by a multidisciplinary team. However, NHS pathways often suffer long waiting times (Russell et al., 2022; Rutherford et al., 2016). This leads some people to pursue a diagnosis through the private sector (Harmens et al., 2022). Unlike a diagnosis via the NHS, a diagnosis sought from a private practitioner is not routinely fed back to general practitioners (GPs), instead being given to the person for them to use as they wish (Russell et al., 2022). This means that diagnosis prevalence estimates typically underestimate the true occurrence of adult autism diagnosis when based only on GP data (Underwood et al., 2022). That said, the incidence of privately acquired autism diagnoses going unrecognised by primary care services has yet to be quantified and is underexplored with respect to its wider implications for support seeking and support provision. Even so, if NHS providers are not aware of a diagnosis obtained via private assessment, the person may experience variable interactions with NHS healthcare systems due to, for instance, reasonable adjustments within NHS services being overlooked for the person thereby risking their service disengagement (Hashmi & Davidson, 2021).

More positive outcomes have been associated with a clinically confirmed adult autism diagnosis with respect to it facilitating increased self-directed compassion and acceptance (Hearst, 2015; Powell & Acker, 2016; Wilson, Thompson, Rowse, Smith, et al., 2023). Nonetheless, it would be wrong to suggest that being given a diagnosis always charts a positive pathway for an individual. Some autistic adults described encountering negative, pessimistic or mixed feelings following their diagnosis, which for some individuals lead to a depressive episode (Huang et al., 2022a; Lewis, 2016; Powell & Acker, 2016). The emphasis on the emotional content of a diagnosis highlights the psychological adjustment that follows a diagnosis. This may involve an individual reconfiguring their self-identity to integrate their autistic identity into their sense of self (Lilley et al., 2022), and reinterpreting or making sense of their life experiences leading up to the point of diagnosis such as a sense of ‘not fitting in’ (Crane et al., 2018; Stagg & Belcher, 2019). Peer support has been found to be effective in helping to elicit positive adult outcomes, with participation contributing to the development of a positive autistic identity (Crompton et al., 2022). Holding a positive autistic identity has been linked to reduced depressive symptoms (Cage et al., 2018; K.Cooper et al., 2017). Positive in-group identification may therefore help to explain why receiving a diagnosis, albeit later in life, can have a positive impact on well-being that is anchored in a view of the self that is not only accepting and compassionate but also takes pride in being autistic (Leedham et al., 2020; Wilson, Thompson, Rowse, Smith, et al., 2023). In this sense, an autism diagnosis can be empowering for the individual by offering them a new framework through which to view themselves in a more positive light.

While an autism diagnosis can have a positive impact on a person’s sense of self, autistic adults are subject to poor outcomes, including high risk of unemployment (Office for National Statistics, 2021), poor mental health (Eaves & Ho, 2008), increased inpatient admission (NHS Digital, 2021) and premature mortality (Hirvikoski et al., 2016). Such outcomes may be explained by unmet support needs, commonly arising due to inadequate post-diagnostic support provision and, where offerings do exist, insufficient information about support options and providers (Huang et al., 2022b; Jones et al., 2014; Lewis, 2016; Raymond-Barker et al., 2018). Jones et al. (2014) reported that 41.9% of their sample of 128 autistic individuals, all of whom had received their diagnosis in adulthood from a UK service, were offered no form of post-diagnostic support. Of the different stages of the diagnostic process, i.e. the pathway from assessment and diagnosis through to the support received thereafter, post-diagnostic support received the lowest satisfaction ratings with only 22.6% of participants reportedly satisfied with support offerings. Specifically, participants in this study requested (1) counselling, (2) social skills training and (3) support groups to be available post-diagnosis.

Counselling and support groups highlight the need for more mental health support which speaks to the higher than average prevalence of co-occurring mental health problems among autistic adults (Hollocks et al., 2019). Recently published studies have identified areas of support that remain a concern for autistic people and their families. These include mental health support provision (Crane et al., 2019); housing, transportation and employment-related support (Tint & Weiss, 2018); and help to secure reasonable adjustments within workplace and healthcare settings (R.Cooper & Kennady, 2021; Haydon et al., 2021). In addition, among clinicians, a lack of confidence in supporting autistic adults is highlighted, particularly for GPs (Unigwe et al., 2017).

With widespread recognition that post-diagnostic support requires improvement, efforts need to be made to establish what entails better post-diagnostic support. One way to approach this is through the Delphi method which describes an iterative process of surveying individuals to arrive at a group consensus (Linstone & Turoff, 1975). The Delphi method is commonly used to facilitate decision-making within health-related research (de Meyrick, 2003; Hasson et al., 2000; Niederberger & Spranger, 2020). The Delphi method can help identify convergence within group opinion. In this respect, the Delphi method produces an understanding of which views are consistently held by community members.

A recently published Delphi study conducted by Wigham et al. (2023) identified 11 consensus statements describing optimal post-diagnostic support for autistic adults from clinicians’ perspectives. Key indicators of optimal support included an additional follow-up meeting 2–4 months after the initial diagnostic feedback meeting; support available in one-to-one and group-based settings; and multidisciplinary teams that include occupational therapists and speech and language therapists, or at the very least other professionals with expertise in autism. While the clinician’s perspective on consensus priorities provides valuable insight into what might be feasible within the confines of clinical practice, it is important that consensus is also sought from autistic adults to ensure the effective design of services.

In the present study, we investigated autistic adults’ priorities for post-diagnostic support. For this, recently diagnosed autistic adults in the United Kingdom devised post-diagnosis support priorities in a four-round modified Delphi study. Traditionally, post-diagnostic support services have been designed and commissioned by non-autistic professionals. This risks services not meeting the needs of the individuals they are trying to serve. The aim of this study was to provide a bank of consensus priorities generated by autistic adults that provides a clear evidence base outlining what autistic adults want from post-diagnostic support. These priorities can then feed into the development of clinical recommendations for support and more optimal service provision.

## Method

### Participants

Autistic adults were invited to contribute to the modified Delphi via the mailing lists of public-sector and third-sector organisations and social media. The invitation was to participate in steering group workshops and/or online questionnaires. Eligibility criteria were that participants must have received their autism diagnosis aged 18 and over from a UK diagnostic service, within the last 10 years.

The questionnaire was distributed in four rounds. Forty-three participants completed the first questionnaire aiming to establish what autistic adults want from post-diagnostic support. Ages ranged from 19 to 60 years (M = 38.71; SD = 12.46). At the point of diagnosis, ages of participants ranged between 18 and 57 years (M = 35.66; SD = 11.90). Respondents who completed the first questionnaire were then invited via email to participate in subsequent questionnaires. Of the initial 43 respondents, 42, 40 and 26 participants completed questionnaires two, three and four, respectively.

For the fourth round, we acquired an additional 113 responses by advertising to autistic adults located in the United Kingdom on Prolific (https://www.prolific.co/), which supplemented the 26 responses collected from the original sample. The fourth-round ages ranged between 19 and 67 years (M = 36.60; SD = 11.22); ages at diagnosis ranged between 18 and 66 (M = 33.12; SD = 10.74). To improve the representativeness of our sample, we specifically targeted individuals who identified as ‘non-White’ and were aged 50 years and over in our recruitment. Of the 139 responses, we acquired 21 responses from individuals identifying as ‘non-White’ and 26 responses from individuals aged 50 years or older.

The breakdown of demographic variables for the samples in rounds one and four are provided in Table 1.

**Table 1.** Demographic characteristics of questionnaire respondents.

Characteristic	Round 1		Round 4	
	*n*	*%*	*n*	*%*
Gender				
Female	24	56	85	61
Male	11	26	43	31
Non-binary	5	12	5	4
Gender non-conforming	1	2	4	3
Questioningª	1	2	0	0
Transgender	0	0	2	1
Prefer not to say	1	2	0	0
Ethnicity				
Black	1	2	8	6
Asian	1	2	3	2
White	33	77	118	85
Mixed heritage	5	12	10	7
Arabª	1	2	0	0
Prefer not to say	2	5	0	0
LGBTQ + community member				
Yes	16	37	57	41
No	22	51	73	53
I am not sure	1	2	9	6
Prefer not to say	4	9	0	0
Existing mental and/or physical health condition				
Yes	28	65	107	77
No	9	21	23	17
I am not sure	2	5	9	6
Prefer not to say	4	9	0	0
Employment				
Full-time caregiver	2	5	3	2
Employed	18	42	72	52
Self-employed	2	5	12	9
Supported employment	1	2	0	0
Unemployed	8	19	29	21
Student	4	9	19	14
Retired	1	2	3	2
Voluntary work	1	2	0	0
Economically inactiveª	0	0	1	1
I am not sure	2	5	0	0
Prefer not to say	1	2	0	0
No answer	3	7	0	0
Highest educational level				
School-leaving qualifications	4	9	17	12
College qualifications	9	21	30	22
Foundation degree	1	2	3	2
Undergraduate degree	25	58	55	40
Postgraduate degree	0	0	33	24
Prefer not to say	1	2	1	1
No answer	3	7	0	0

The table lists only the response options that participants selected.

ª Response option added by participants after they selected the ‘Other’ option.

### Procedure

Discussion during the first of three steering group workshops (see ‘Community involvement statement’ section) enabled researchers to generate an initial list of 48 questionnaire items that, in autistic adults’ views, should be included in post-diagnostic support. Each questionnaire item was generated from a point made by one of the steering group members; none were generated from preconceived ideas of the non-autistic researchers.

The 48 questionnaire items were then used to generate an online questionnaire to ask for autistic adults’ views on what post-diagnostic support should comprise. For each item, participants were asked to rate on a five-point Likert-type scale the extent to which they agreed that the statement represented an important element that should be included in post-diagnostic support, from ‘1’ (strongly disagree) to ‘5’ (strongly agree), thus facilitating engagement with each item. Items that received low consensus agreement (< 40%) were modified before presentation in subsequent rounds. Open-ended text boxes were included for participants to suggest modifications to existing items and to identify any missing priorities. The first questionnaire and subsequent versions were created using Google Forms or Qualtrics and accessed via an online link. The questionnaires have been uploaded to the OSF repository (https://osf.io/h3txq/).

As mentioned, questionnaire respondents made suggestions for adding and modifying items via the open-text comment boxes. All missing support priorities that participants identified were added to the second and third iterations of the questionnaire. Suggestions for modifying the wording of items were reviewed in relation to one another. If convergence emerged then questionnaire items were modified accordingly. In workshops two and three, corresponding with Delphi rounds one and two, input was gathered from steering group members as to the exact rewording of existing items that warranted modifying.

**Figure 1.**
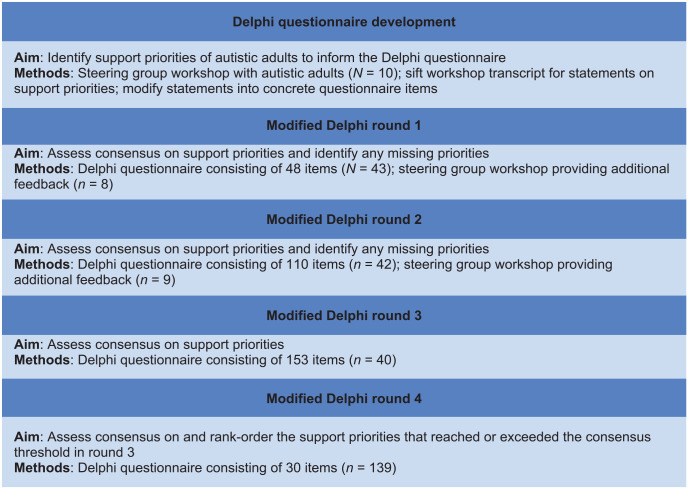
Flowchart of modified Delphi study design.

A fourth Delphi round sought autistic adults’ opinions on the final list of support priorities, identified via the prior three Delphi rounds. This helped to ensure the order of importance and wording of priorities aligned with perspectives from the wider autistic community. See Figure 1 for a study design outline.

The research team organised the full set of 153 questionnaire items into six emerging topics to provide some structure to the questionnaire for participants: delivery of support (*n* of items = 33); emotional and psychological support (*n* of items = 26); person-centred support (*n* of items = 28); supporting relationships (*n* of items = 12); practical support (*n* of items = 39); and support understanding autism (*n* of items = 15).

Remuneration payments in line with the National Institute for Health and Care Research (NIHR) guidance were made to participants. Participants provided informed consent via an online check box. The study was approved by the University of University of Sheffield’s Ethics Committee (approval number: 045002).

### Data analysis approach

A threshold of ⩾80% of respondents scoring an item a ‘4’ (agree) or ‘5’ (strongly agree) was used to appraise consensus. This threshold criterion was determined based on other Delphi threshold values in autism research that have ranged from 50% to 80% (e.g. see Higgins et al., 2021; Howell et al., 2022), aiming for the upper limit.

As is the common Delphi procedure (Ali et al., 2018; Salmon & Tombs, 2018; Zervogianni et al., 2020), mean scores of items were used to rank-order support priorities in the round four Delphi questionnaire. Standard deviation and coefficient of variation (CV) were used to evaluate the degree of score dispersion around the mean. Percentages for the CV were appraised based on English and Kernan’s (1976) guidelines, which suggest the degree of consensus is good if 0% < CV ⩽ 50%, less than satisfactory if 50% < CV ⩽ 80%, and poor if CV > 80%.

### Community involvement statement

One of the study authors is autistic. A steering group of 10 autistic adults co-designed the study across a series of three workshops. Their contribution focussed on 1) acquiring feedback on the study design; 2) generating ideas on the elements that should be included in post-diagnostic support; and 3) reconsidering and rephrasing existing questionnaire items requiring modification. Steering group workshops were facilitated by a relative of an autistic adult. Vouchers were distributed to attendees whose work equated to approximately three half day’s activity. Anonymised data on demographics and information on the recruitment strategy for the steering group is available in the open-access data repository (https://osf.io/h3txq/).

Researchers also engaged with parents and carers of autistic adults along with clinicians and commissioners working in the field of autism, prior to running the modified Delphi. Together, they convened in a workshop where they reviewed the research agenda, which was perceived as being of direct relevance and usefulness to the different stakeholders.

A dissemination event was held online in July 2022 to share the study results with autistic people, caregivers, clinicians and service commissioners. Following this, a lay report outlining the study findings was circulated among the autistic community and within NHS and social care settings. This report was accompanied by an Easy Read version that was reviewed by an autistic adult with expertise in producing Easy Reads. They received vouchers to thank them for the time and effort this involved.

## Results

### Consensus results

Of the 153 support priorities identified and rated by participants over the four Delphi rounds, 24 priorities (16%) met or exceeded the consensus threshold with 80% of participants being in agreement or strong agreement that these items represented important elements to be included in post-diagnostic support. Table 2 lists the support priorities that achieved consensus in order of mean rankings and includes measures of score dispersion, specifically standard deviation and CV. According to English and Kernan’s (1976) guidelines for interpreting CV percentages, 20 support priorities had a good degree of consensus, while four had a less than satisfactory degree. This suggests that mean scores, based on which support priorities were ranked, closely represent the majority of participants’ scoring. A complete list of the 153 support priorities identified by participants can be found in the study’s data repository, as they were ranked in round three of the modified Delphi (https://osf.io/h3txq/).

**Table 2.** Support priorities that reached consensus ranked by mean score from the highest to the lowest priority.

Rank	Support priority	Topic	Consensus (%)	Mean	Standard deviation	Coefficient of variation (%)
1	Access to support irrespective of where I live	1	98	4.76	0.51	41^a^
2	Access to professionals with specialist up-to-date training on autism	1	95	4.71	0.61	47^a^
3	Access to mental health professionals with specialist knowledge of autism	1	94	4.69	0.65	49^a^
4	Includes support to process the impact of a late diagnosis	2	90	4.60	0.77	55
4	Support takes into account my communication and contact preferences	3	96	4.60	0.62	44^a^
6	Includes an individualised support plan that is tailored to my needs	3	95	4.53	0.62	42^a^
7	Opportunity to access services when I need them	3	97	4.49	0.66	44^a^
7	Services designed in collaboration with autistic people	1	89	4.49	0.76	50^a^
9	Help with autistic fatigue	2	89	4.46	0.76	50^a^
9	My support plan would include personalised coping strategies	3	92	4.46	0.67	44^a^
11	Help accessing support, with social anxiety	1	94	4.44	0.73	47^a^
12	My support plan would take a holistic approach that looks at the whole person	3	90	4.42	0.76	48^a^
13	One-to-one support	2	89	4.41	0.85	53
14	My support plan would take into account my coexisting conditions (if appropriate)	3	89	4.37	0.75	46^a^
15	Help with financial aid for specialist equipment e.g. noise-cancelling headphones	5	89	4.36	0.85	52
15	Begins at a point that feels right for me post-diagnosis	3	89	4.36	0.71	43^a^
17	Includes follow-up appointments with professionals	1	89	4.34	0.76	46^a^
17	My support plan would take into account past trauma (if appropriate)	3	89	4.34	0.85	51
19	Help with self-empowerment	2	89	4.31	0.77	46^a^
20	The option to access support immediately post-diagnosis	1	92	4.30	0.73	43^a^
21	My support plan would take into account my age	3	85	4.29	0.79	46^a^
22	Inclusive autism-specific services	1	89	4.26	0.70	40^a^
23	Help to develop a positive autistic self-identity	2	83	4.18	0.88	48^a^
24	Help with accessing healthcare	1	88	4.17	0.80	44^a^

Topic 1: Delivery of support; Topic 2: Emotional and psychological support; Topic 3: Person-centred support; Topic 4: Supporting relationships; Topic 5: Practical support; Topic 6: Support understanding autism.

a Good or better levels of consensus.

### Questionnaire topics

As Table 2 illustrates, the 24 priorities reaching the consensus threshold of ⩾80% of respondents scoring an item a ‘4’ or ‘5’ were clustered within four of the six topics. The largest proportion of priorities were grouped within ‘Delivery of support’ (*n* of items = 9) and ‘Person-centred support’ (*n* of items = 9), a smaller proportion were grouped within ‘Emotional and psychological support’ (*n* of items = 5), and only one priority was grouped within ‘Practical support’.

## Discussion

It has been well documented that the provision of post-diagnostic support for autistic adults in the United Kingdom is both geographically inequitable and generally considered inadequate (e.g. Crane et al., 2018; Jones et al., 2014; Scattoni et al., 2021). The current study sought to establish autistic adults’ consensus priorities for post-diagnostic support. Participants ranked *access to support where they live*, *training of professionals*, *support to process the impact of a late diagnosis*, *use of their preferred mode of contact* and *an individualised support plan* as their key priorities. Identifying and understanding these and other priorities has the potential to improve the quality, consistency and range of support. Using a modified Delphi method, 153 support priorities were identified by autistic adults. Of these, 24 priorities reached or exceeded the consensus threshold of 80% of respondents being in *agreement* or *strong agreement* that the item describes an important element of post-diagnostic support. Below each topic into which these 24 priorities cluster is discussed in turn.

### Delivery of support

Delivery of support, defined as the way through which support services and resources are accessed by autistic adults, encompasses the highest overall ranking support priority of the 24 items, ‘*Access to support irrespective of where I live*’. Evidence suggests that the assessment process undergone to get an autism diagnosis can be highly variable, as can the support received thereafter, resulting in a ‘postcode lottery’ with respect to the provision of formal post-diagnostic support (Beresford et al., 2020; Beresford & Mukherjee, 2023; McLinden & Sedgewick, 2023; Wilson, Thompson, Rowse, et al., 2023). This demonstrates the potential benefits of implementing national guidance (e.g. National Institute for Health and Care Excellence (NICE), 2012) that remains only partially and variably put into effect (Wigham et al., 2023), but promises to standardise support practices.

The second and third highest ranking of the 24 support priorities were ‘*Access to professionals with specialist up-to-date training on autism*’ and ‘*Access to mental health professionals with specialist knowledge of autism*’. The need for highly skilled and knowledgeable professionals evidenced in the two statements concurs with the finding of Wigham et al.’s (2023) study. In this previous Delphi, the majority of clinicians endorsed expertise in autism as a crucial component in delivering effective post-diagnostic support, providing a clear indicator of clinician readiness for and receptiveness to training. Hence, rather than clinician willingness, the costs incurred by training seem to be a more significant obstruction to training access with funding for transferring and perpetuating autism knowledge and expertise via training being demonstrably lacking (Beresford et al., 2020; Wigham et al., 2023). Nicolaidis et al. (2015) highlighted the importance of allocating available funds to training programmes that not only impart knowledge of the characteristics of autism but also address attitudes, skills and behaviours required for providing effective healthcare for autistic adults (Nicolaidis et al., 2015). Unigwe et al. (2017) corroborated this, evidencing a high level of basic knowledge about autism among GPs, but limited confidence in managing autistic patients’ needs.

Other priorities that participants agreed were important included ‘*The option to access support immediately post-diagnosis*’, ‘*Help with accessing healthcare*’ and ‘*Help accessing support, with social anxiety*’. With each priority advocating for access to support or help to make services more accessible, these provide the impetus for commissioners and service providers to deliver accessible support. Camm-Crosbie et al. (2019), after surveying 200 autistic adults about their experiences of mental health support, found that care was not always felt to be tailored to individual needs. Similarly, Crane et al. (2019) reported concurring findings for young autistic adults seeking mental health support. ‘*Services designed in collaboration with autistic people*’, endorsed as a priority by our sample, could help to overcome this barrier to support access through the co-development of tailored support. However, it is clear that further resources are required to meet the needs identified.

Value was also recognised by participants in support that ‘*Includes follow-up appointments with professionals*’ and ‘*Inclusive autism-specific services*’. The first of these is consistent with the NICE clinical guidance and Wigham et al.’s (2023) clinician consensus Delphi study, both of which endorse a follow-up appointment post-diagnosis. Discussion with autistic adults concerning the number of follow-ups and the time period over which they would like to see these offered could benefit proceedings post-diagnosis. Related to the second of the two priorities, the NICE guidance set out a recommendation for ‘Specialist Autism Team’ (SAT) provision in local authorities (NICE, 2012), again highlighting convergence between the preferences of our participants and the NICE clinical guidance. The role of SATs within local communities is to help ensure that healthcare services are suitable for supporting autistic adults. In implementing the NICE clinical guidance, autism-specific services have subsequently been realised in the form of SATs in 18 localities across England (Beresford et al., 2020).

### Emotional and psychological support

‘*Help to develop a positive autistic self-identity*’ and ‘*Help with self-empowerment*’ both emerge as support priorities in this topic. Previous findings suggest that a positive autistic identity may help to challenge a pre-diagnosis identity that viewed the self as intrinsically flawed and as having to remain camouflaged (Leedham et al., 2020). For autistic women, however, it has been recognised that shifting identities can bring with it the unique challenge of disentangling their ‘authentic self’ from their ‘masking self’, a process that for some people can lead to anxiety and depression (Bargiela et al., 2016). Moreover, women reported encountering further difficulties, fearing how others may react to them showing their ‘authentic selves’ (Leedham et al., 2020). While masking is particularly pertinent to autistic women’s experiences, the phenomenon of masking is not female-specific (Hull et al., 2017; Livingston et al., 2019), hence it is unlikely that such challenges are unique to women. Not only does this shed some light on autistic adults’ desire for more support in developing a positive self-identity and self-empowerment, but it also provides an idea of the type of support that may constitute ‘*support to process the impact of a late diagnosis*’, the highest-ranking priority, namely help to address issues related to adopting a new autistic identity late in life, which may involve processing the impact of years spent masking.

‘*Help with autistic-fatigue*’ is the second highest ranked priority in the topic, with autistic-fatigue being defined as a response to stressors encountered daily by autistic individuals in a world that is skewed towards neurotypical norms. It follows that one reason for some autistic people wanting help with autistic-fatigue may centre on the pressure of having to act in a way that seemingly complies with neurotypical norms and emergent societal standards. Indeed, stress arising from constant pressure to mask autistic traits, motivated by a desire to ‘fit in’, has been identified by autistic adults as one of the key causes of autistic fatigue (Hull et al., 2017), and masking has been linked to exacerbated mental health difficulties (Bargiela et al., 2016; Cage & Troxell-Whitman, 2019). This suggests that help with autistic-fatigue, which would seemingly be a difficult topic to advise on without providing help with the negative impact of masking, would be of benefit to mental health outcomes for autistic adults. Further exploration in relation to autistic fatigue is needed to establish what exactly this help should involve and whether it could be delivered through, for instance, an autistic-led programme. With ‘*One-to-one support*’ having been identified as another support priority, this also constitutes a potential mode of delivery for help with autistic-fatigue that is worthy of inquiry.

### Person-centred support

Participants made a clear request that post-diagnostic support should ‘*Include an individualised support plan that is tailored to my needs*’ and there was consensus on a range of key elements that should be included in this. Specifically, participants agreed that ‘*My support plan would take a holistic approach that looks at the whole person*’, ‘*My support plan would take into account my coexisting conditions (if appropriate)*’ and ‘*My support plan would include personalised coping strategies*’. This dovetails with work emphasising autistic people’s desire for diagnostic services that adopt a holistic approach (D’Arcy et al., 2021). A support plan taking into account coexisting mental and physical health conditions not only exemplifies a holistic approach but also reflects the NICE (2012) clinical guidance which recommends due consideration is given to the impact and management of comorbidities. Importantly, a support plan that delivers personalised coping strategies would need to consider the possible impact of concurrent disorders. With interventions, for example, cognitive behavioural therapy, for mental health disorders being adapted for autistic people (Spain & Happé, 2020), strategies in a support plan could potentially incorporate those from viable psychotherapeutic interventions.

Consideration of past experiences was also deemed important, ‘*My support plan would take into account past trauma (if appropriate)*’. The trauma criterion resonates with previous findings suggesting that autistic people are highly vulnerable to trauma following events, such as sustained bullying (Rumball et al., 2020). Building on this line of theorising, while Rumball et al. (2020) highlight the importance of assessing trauma and PTSD symptomology in autistic adults, findings in the current Delphi study extend this to suggest that psychological help for trauma-exposed individuals should be incorporated into tailored support plans. Another key factor of importance was age, ‘*My support plan would take into account my age*’, which resonates with a range of previous literature that discusses how autistic adults often feel they need to come to terms with the experience of receiving a diagnosis relatively late in life, the result of which can be a range of negative emotions, such as feelings of grief on behalf of their former self (Huang et al., 2020; Leedham et al., 2020). This reflects the kinds of unique challenges associated with being diagnosed in adulthood rather than childhood. Acknowledging such differences highlights how child-centred autism services may not be fully equipped to offer professional support for the challenges newly diagnosed adults may face.

Furthermore, there was consensus on support delivery being tailored to the individual, ‘*Support takes into account my communication and contact preferences*’, and there was desire for support to be arranged/accessed within a timeframe that was right for the individual, ‘*Begins at a point that feels right for me post-diagnosis*’ and ‘*Opportunity to access services when I need them*’. The clinical implications that can be inferred here are that people may be amenable to support and services via different modes of delivery at times other than the point of diagnosis. This suggests that clinicians may do their best to consult patients to identify preferences for how support is delivered. Furthermore, services should consider periodically assessing a person’s readiness to engage with support offerings, especially if support is initially turned down by the individual. Importantly, the latter of the statements indicates clear consensus for ‘step-on step-off’ services whereby services can be accessed as many times as they are needed, without a new referral, by an individual post their original referral. This approach has previously been suggested as valuable in autism services (Wigham et al., 2023).

### Practical support

The single priority identified in this topic was ‘*Help with financial aid for specialist equipment e.g. noise-cancelling headphones*’. In their literature review of financial costs associated with autism, Rogge and Janssen (2019) identify specialist equipment as an expense that is frequently borne by individuals themselves or their families, which can become a significant financial burden. Shouldering such costs may be especially challenging for autistic adults who face disproportionate rates of unemployment, with data from the Office for National Statistics (2021) suggesting only 22% of autistic people are in employment as compared with 80% of non-disabled people. Nevertheless, even when in employment, autistic adults reported being without appropriate accommodations in the workplace, such as specialist equipment (Davies et al., 2022). This suggests that, despite a high proportion of our sample being in employment (49% and 60% in rounds one and four respectively) compared to reported rates for autistic adults, a lack of successfully implemented workplace accommodations may still leave participants under considerable financial strain from costs incurred by the purchase of specialist equipment. Hence, this priority suggests value in research identifying the importance of implementing workplace adjustments and allocating funds sufficiently.

### Strengths and limitations

The sample size of rounds one, two and three of the current modified Delphi could be seen as a limitation to generalisability. However, given that the purpose of a Delphi is to arrive at expert consensus, the sample size used for rounds one, two and three was already larger than is generally seen in the literature (Okoli & Pawlowski, 2004). Nonetheless, efforts were made to acquire a larger sample in the fourth round to assess whether the priorities identified would be endorsed by the broader community of autistic adults. Efforts were made to recruit a more diverse sample with respect to ethnicity and age in the fourth round. Fifteen percent of the sample identified as non-White, nearing the national figure of 18% that constitutes ethnic groups other than White in England and Wales, according to 2021 census data (Office for National Statistics, 2022a). Nineteen percent of our sample were aged 50 years or older. A recent systematic review highlighted that only 0.4% of published autism studies included a cohort of adults aged 50 years or older (Mason et al., 2022); thus, we were pleased to be able to report some data from this very underrepresented age bracket. However, only 1% of our sample were aged 65 years or over, as compared to 19% of the total population in England and Wales (Office for National Statistics, 2022b). Hence, it will be important that future work seeks to specifically acquire input from older adults on post-diagnostic support needs.

An aspect of this modified Delphi that departed from standard methodology was that data from rounds one and two were not seen by participants in subsequent rounds. With many items being added across each of the first three Delphi rounds, had we presented these data, this would only have been applicable to some of the questionnaire items. We were concerned that presenting items in this way could have been confusing for participants particularly given that consistency within questionnaires is highly valued by autistic adults (Nicolaidis et al., 2015; O’Nions et al., 2018; White et al., 2021). However, in the fourth round, with data on consensus acquired for all 30 items in round three, these data were presented to respondents. This enabled participants to generate a response building on their knowledge of scores acquired in the previous round.

A second key aspect of this modified Delphi that distinguishes it from a conventional Delphi survey design was its inclusion of a steering group of autistic adults who helped to determine the study methodology. Typically, studies using a classical Delphi technique remove items that reach consensus, only retaining items that fall below the consensus threshold for revision in subsequent iterations of the questionnaire. However, to ensure a fair priority-setting process, we modified the approach, retaining items for additional consideration in subsequent rounds regardless of whether consensus was achieved. This was informed by the views of steering group members and by similarly modified Delphi studies that adapted procedure to eliminate the possibility of remaining items receiving inflated ratings of importance (Shattuck et al., 2018; Trevelyan et al., 2015). The co-produced nature of the study is a strength of this piece of research as it extended more traditional Delphi processes that rarely focus on participatory methods, providing an example of meaningful community involvement that others could emulate.

The questionnaire item development being autistic-led enabled this modified Delphi study to contribute a unique perspective to the literature on understanding what constitutes optimal post-diagnostic support. In this sense, as has been done in other studies (e.g. Higgins et al., 2021), broadening a traditional understanding of ‘experts’ within Delphi research beyond those with clinical expertise to include those with lived experience is a step towards delivering inclusion in autism research. Extending this modified Delphi through further refinement with a nationally representative sample of autistic adults, or a sample in which people from traditionally underserved groups are over-represented, would help to ensure that the findings are broadly generalisable. Indeed, with our sample excluding those experiencing digital poverty, being highly educated and a high proportion being in work, whether the findings are applicable to other autistic adults needs to be determined. In this respect, the current study provides a large bank of support priorities that constitute a start rather than an endpoint in the process of determining what autistic adults want from post-diagnostic support.

### Future recommendations

This clear evidence base now presents the opportunity for commissioners, designers of services and researchers to begin to improve the quality of post-diagnostic support for autistic adults, who are currently very poorly served. Work investigating barriers to implementing autistic adults’ priorities would help to facilitate the development of services conducive to autistic peoples’ requirements. Inquiry into autistic adults’ support priorities in different contexts is also warranted. Settings for this line of investigation might include countries and care systems other than the United Kingdom and NHS, or supported living and residential support units. This would enable a better understanding of the ways that autism-related support needs intersect with demographic dimensions, such as nationality and support needs. In this sense, this may provide a research approach that derives context-specific recommendations that can be used to tailor support offerings to autistic people post-diagnosis.

## Conclusion

The current study provides consensus-driven guidance on autistic adults’ key priorities for post-diagnostic support. It is clear that an approach involving local support provision and knowledgeable professionals would be valued by autistic adults. Many features evident in the current list of priorities were also identified in the 11 consensus priorities for autistic adult post-diagnostic support agreed upon by clinicians (Wigham et al., 2023). However, the current research also identified a range of specific features that autistic adults feel should be incorporated in post-diagnostic support not covered by previous research, such as help to address the impact of a late diagnosis, takes into account communication preferences, includes an individualised support plan tailored to my needs, should be designed in collaboration with autistic people, includes help with autistic fatigue and includes personalised coping strategies. Hence, the list of 24 priorities identified in the current study provides a range of practical elements that autistic adults see as essential to post-diagnostic support. This study therefore provides new insights into the perspectives of autistic adults that extend previous understandings of what constitutes optimal post-diagnostic support. This emphasises the importance of autistic adults being given the opportunity to have their say when it comes to determining what support and services are available to them following their diagnosis. By no means do all of the priorities indicate the need for additional resources, although this is a necessity if post-diagnostic support for autistic adults is to become fit for purpose. Taken together, the consensus priorities identified by autistic adults in this modified Delphi study provide the basis for making significant improvements to post-diagnostic support for autistic adults.
